# Effect of a Clear Communication Training on Medical Residents’ Use of Jargon in Written Messages to Patients

**DOI:** 10.3390/healthcare14101374

**Published:** 2026-05-18

**Authors:** Cliff Coleman, Chelsea Lin

**Affiliations:** 1 Department of Family Medicine, Oregon Health & Science University, 3181 SW Sam Jackson Park Road, MC FM, Portland, OR 97239, USA; 2 Center for Ethics in Health Care, Oregon Health & Science University, 3181 SW Sam Jackson Park Road, MC UHN-86, Portland, OR 97239, USA; 3 Department of Anesthesiology and Critical Care, Hospital of the University of Pennsylvania, 3400 Spruce Street, Suite 680 Dulles, Philadelphia, PA 19104, USA; lin_chelsea@outlook.com

**Keywords:** doctor–patient communication, clear communication, written communication, plain language, jargon, health literacy, training, curriculum

## Abstract

**Highlights:**

**What are the main findings?**

- Medical residents use jargon in personalized written communication with patients.
- Residents’ use of unexplained jargon in written messages to patients decreased during 3 months of follow-up after a brief training on health literacy and clear communication.

**What are the implications of the main findings?**

- Participation in a skill-building workshop that includes rewriting messages to improve understandability for patients may contribute to the development of plain language habits regarding unexplained jargon use among resident physicians.
- To our knowledge, this is the first study to quantify physicians’ use of jargon in written communication with patients, and the first to show a reduction in such jargon associated with training.

**Abstract:**

**Background/Objectives:** Despite recommendations to use plain language for better outcomes in clinical care, physicians and other health care professionals frequently use unexplained medical jargon when communicating with patients. Few studies have examined the use of jargon in personalized written communication with patients. We evaluated the effects of a clear communication curriculum on medical residents’ use of unexplained jargon in written health-related messages sent to patients through a secure electronic patient portal. **Methods:** We retrospectively collected messages sent by first-year family medicine residents to patients during the 3 months before and 3 months after completion of a curriculum on health literacy and clear communication. Two researchers independently coded jargon in messages according to Pitt and Hendrickson’s taxonomy. Messages with ≥100 words were analyzed for readability using the Flesch-Kincaid Grade Level score. Rates of jargon use and mean readability were compared using *t*-tests. **Results:** Nine first-year residents produced a total of 421 messages in the pre- and post-intervention periods. The rate of unexplained jargon decreased from 7.2 to 6.3 jargon terms per 100 words following the training (*p* = 0.05), a 12.5% reduction, equivalent to nearly one fewer jargon term per message. Jargon rates did not differ by jargon subtype, and average readability did not change. **Conclusions:** This is the first known study to quantify physicians’ use of jargon in personalized messages written to patients. Jargon use was common. Exposure to the curriculum was associated with borderline significantly reduced use of unexplained jargon in the 3 months following training.

## 1. Introduction

Effective communication between physicians and patients is essential to delivering high-quality, safe, efficient, patient-centered, satisfying, and equitable health care. Yet “effective” patient–provider communication has not been clearly operationalized for medical trainees. Making health information understandable is one important feature of effective communication. To help facilitate understandability in written communication with patients, experts recommend avoiding unnecessary and/or unexplained medical jargon and writing at a 5th–6th-grade readability level, among other best practices [1,2]. Patient-accessible portals provide a secure web-based means for patients to access certain aspects of their electronic health record, and to send and receive written electronic messages (emails) with clinicians involved in their care. In 2024, nearly two-thirds of U.S. adults used a patient portal [3]. Very little is known about the quality of messages sent to patients through such portals, or patients’ ability to read, understand, act on, and benefit from such communications. Understanding and addressing clinicians’ use of unexplained jargon and high grade-level readability in personalized written messages to patients may offer a modifiable means of improving clinician-patient communication.

### 1.1. Jargon

Medical jargon is specialized language or language used in a specialized way with unique meaning in health- or health care-related contexts, and which may be misinterpreted or misunderstood by people less familiar with its use. Physicians use unnecessary and undefined jargon in spoken communication with patients [4,5,6,7,8,9,10,11,12,13]. Although the use of jargon in mass-produced patient education materials is well documented, few studies have examined physicians’ use of jargon in personalized written messages to patients (e.g., print or electronic correspondence, and free-text portions of outpatient visit summaries, emergency department discharge instructions, and hospital discharge summaries). Among 128 adults who had undergone an endoscopic procedure and were sent written information about the results by mail, 46% said they did not understand the results, and 51% complained about the use of “unfamiliar technical and medical jargon” [14].

Patients generally have an inadequate or incomplete understanding of jargon [15,16,17,18,19,20,21,22], such as terms like “fracture” [16] and “benign” [19], even when they report being familiar with the term [15]. Only 12% of U.S. adults have proficient health literacy skills [23]. Lower health literacy—one’s ability to get, understand, and use health information [24]—may make jargon even more difficult to understand. When used in spoken communication with patients and caregivers, jargon is rarely defined [5,6,8,9], which impedes understanding [25]. Replacing jargon terms with non-jargon plain language alternatives in written consultation letters from specialists to primary care providers was associated with improved understanding and ratings of physicians’ professionalism by patients who read their “translated” letters [26].

### 1.2. Readability

Experts recommend that health information be written at a 5th–6th-grade level in order to make materials accessible to the majority of U.S. adults [1,2]. Few studies have examined the readability level of personalized messages written to patients. One small study examining the physician-entered portions of After Visit Summaries written by internal medicine residents found that a brief training followed by individualized feedback was associated with an increase in the percentage of physicians writing at or below a 6th-grade readability level [27]. The authors of that study, however, did not report on the statistical significance of their results. Several readability formulas are commonly used to estimate the education level required to read and understand a written text. For example, the Flesch-Kincaid Grade Level is the most commonly used readability formula in health communication research [28]. Readability formulas, however, do not identify jargon, and lower readability scores do not ensure understandability, as one can write short sentences with only one- and two-syllable words that are nonetheless hard to understand.

Patients with both higher and lower health literacy show improved knowledge when appropriate written materials are used [29]. Despite recommendations to use clear communication practices when writing to patients [1,2], resident physicians get little training in clear written communication [30,31], and little is known about the effectiveness of interventions to improve clear written communication for patients. We sought to evaluate the impact of a written communication training on medical residents’ use of jargon in personalized electronic messages sent to patients through a secure patient portal. We hypothesized that residents would use less undefined jargon overall after completing a curriculum on health literacy and clear communication best practices. We also hypothesized that specific subtypes of jargon, such as technical jargon and abbreviations/acronyms, as well as the grade level readability demands of written messages, would decrease as well.

### 1.3. Curriculum

Family medicine residents at our institution receive mandatory training on health literacy and clear communication, consisting of four 2 h sessions during the first 7 months of residency year one. An earlier version of this curriculum was shown to produce long-term improvements in residents’ self-reported knowledge of health literacy and clear communication principles, but only modest self-reported improvements in spoken communication practices [32]. The current competency-guided [2] curriculum outlined in Table 1 uses an episodic/longitudinal design to allow for intentional repetition and distributed learning over time [33].

Part 1 develops foundational knowledge related to health literacy and clear communication. Part 2 builds on this knowledge and includes skills practice in the avoidance of unnecessary and/or unexplained jargon in spoken communication. Part 3 uses an interactive didactic format to explore issues related to numeracy (communicating with numbers and mathematical concepts, such as risk). In Part 4, residents apply elements from the previous sessions in a written communication skills workshop that starts with an interactive didactic presentation on clear writing best practices: (a) writing at a 5th–6th-grade reading level by limiting sentences to 15 words or shorter, using familiar words, avoiding words with three or more syllables, and using the active voice and a conversational tone to reduce formal register; (b) leading with the most important message; (c) emphasizing three key points—what is the main issue, what should be done about it, and why?; (d) using short action-oriented statements; (e) conveying numeric information using low numeracy techniques, such as rounding to whole numbers and using natural frequencies; (f) using the “universal medication schedule” (“morning,” “noon,” “evening,” and “bedtime”) for medication instructions; (g) following principles of easy-to-read design and formatting, such as informative subject headings and bullet lists; and (h) using analogies and examples, while avoiding idioms, metaphors, and euphemisms [1,2]. In the skill-building portion of the session, small groups of three to four residents revise one of several real messages on varying medical topics that were previously sent electronically to patients by former residents in the program. Each of these messages is at least 100 words long to help ensure the accuracy of readability estimates [28] and has had all patient and resident identifiers removed. A variety of readability estimates are presented along with each message, including the Flesch-Kincaid Grade Level, which ranges from 5th to 8th grade for messages used in the activity. Words with three or more syllables are underlined, but jargon is not otherwise identified. Teams are given 10 min to revise their message, with the goal of making it more understandable and more actionable. Participants are advised to use periods at the end of any bullet list items to improve readability scores using automated calculators. Revised messages are reviewed and discussed by the larger group, and the text is then entered live into an online readability calculator with the results displayed for the larger group to see. In 2023–2024, a weather event caused the Part 3 numeracy session to be cancelled. An abridged 40 min version of the numeracy session was incorporated into the Part 4 written communication workshop, shortening the written communication portion to 80 min (Table 1).

## 2. Materials and Methods

### 2.1. Design

We conducted a retrospective cohort study involving all nine first-year resident physicians who entered the Family Medicine Residency Program at Oregon Health & Science University (OHSU) in July 2023, and whose continuity clinic was at one of the three university-affiliated sites in Portland, Oregon. All nine participated in a required longitudinal health literacy and clear communication curriculum (Table 1). The primary outcome of interest was change in the overall amount of unexplained jargon used in written messages sent to patients through a secure patient portal after completing the training. Secondary outcomes included change in the use of jargon subtypes identified using the jargon taxonomy developed by Pitt and Hendrickson [34] and the grade-level readability of messages. The study received an exemption from the Institutional Review Board at OHSU.

### 2.2. Data Collection

We collected all personalized electronic written messages sent by the nine resident physicians in our cohort through our institution’s secure patient portal (Epic MyChart^®^, Epic Systems Corporation, Verona, WI, USA to patients 18 years or older and whose preferred language was English, during the 3 months prior to and 3 months after completion of Part 4 of the longitudinal training intervention, which took place on 4 February 2024 (Table 1). We excluded messages that were sent as attachments to laboratory test results because we felt that these would contain a higher amount of unavoidable jargon. Written messages were anonymized by replacing any individuals’ names or phone numbers with a placeholder, which preserved the original word count of the message. Messages were otherwise included in full, verbatim, without editing or addition of punctuation. Messages sent by patients were not included, and the context of individual messages within conversation threads was not known.

### 2.3. Defining and Operationalizing Jargon

The Pitt and Hendrickson taxonomy [34] is described below. First, however, we describe our strategy for defining and operationalizing jargon in general. There is no consensus definition of medical jargon, and few have proposed operationalized definitions. Links and colleagues [10] (p. 1113) defined jargon as “…any medical terminology which may be unfamiliar to persons without clinical experience,” however, we wanted to allow for a broader set of “words, phrases, or concepts…which might not be fully understood, or may be misinterpreted by the recipient” [2], to allow for terms that are not necessarily “medical terminology,” but may nonetheless cause confusion. For example, “admitted” could mean “put in the hospital” or “confessed.” Moreover, we lacked an operationalizable means for deciding when a health- or health-care-related term should be considered jargon and when it should not. For example, the terms “doctor,” “physician,” “clinician,” and “provider” all carry specialized meanings that differ from one another. Yet, while “doctor” and “provider” likely differ in their population-level familiarity, there is no validated objective means for determining when a given term should be deemed “unfamiliar.” Jargon occurs along a spectrum, including what others have termed “higher-level medical terminology,” that may be less easily understood compared with lower-level jargon [10]. Miller and colleagues [11], using Pitt and Hendrickson’s taxonomy [34], did not count what they called “very simple terms” that are “commonly known” (e.g., “blood pressure”). There is, however, no accepted systematic method for determining the threshold between higher-level and lower-level jargon, nor whether a term is “commonly known.” Wood and Gupta [13] considered medical words to be non-jargon if they appeared on a list of 3000 words expected to be understood by U.S. 4th graders; however, the authors did not validate this approach in their study.

At the individual level, familiarity depends on education, experience, and context. Moreover, familiarity does not necessarily equate to adequate understanding [15]. Furthermore, it is not clear how researchers should determine a threshold for familiarity at a population level, in order to label some health- and health-care-related terms as jargon and others not. Because we could not confidently determine where to subjectively “draw the line” for the purposes of distinguishing jargon from non-jargon, we defined jargon more inclusively as any term with unique or specific significance in a health or health care context. Thus, some seemingly innocuous terms like “doctor,” which are likely not problematic in most instances, were nonetheless included in our ascertainment. Because we applied this broadly inclusive standard consistently to messages in the pre- and post-intervention period, we were able to compare changes in rates of jargon without concern for information bias related to the question of familiarity.

A second issue that has not been adequately addressed in health communication research is how to handle different versions of jargon terms within a given lexeme (family of words). The understanding of words depends on the receiver’s prior knowledge, as well as the context in which the word appears. We are not aware of data indicating that varying forms of a base word (lemma) within a given lexeme are equally understood for medical jargon terms. While Miller and colleagues [11] treated related terms within a given lexeme as a single jargon term, we treated different versions of a word as unique, with the exception of simple differences between singular and plural forms, which we treated as the same. For example, “refer,” “referred,” and “referral” were coded separately, while “referral” and “referrals” were coded as the same.

### 2.4. Coding Medical Jargon

**Figure 1:**
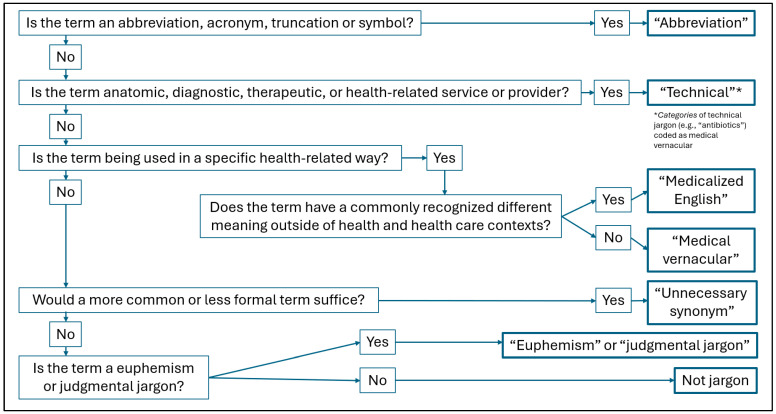
Jargon decision rule used for the seven-category taxonomy [34].

Two trained reviewers read all messages independently and coded jargon according to the seven-category taxonomy developed by Pitt and Hendrickson [34]—technical, abbreviations/acronyms, medical vernacular, medicalized English, unnecessary synonyms, euphemisms, and judgmental jargon—which we chose because it has been used to quantify and classify jargon in spoken encounters [11]. We operationalized the taxonomy with a decision rule developed for our study (Figure 1). Assessments of the first 10 messages were reviewed and discussed informally, showing high interrater agreement on what constituted jargon by our definition (above), and jargon subtype based on the decision rule. Discrepancies were resolved through consensus discussion. Quality control included frequent cross-checking for the presence of a given jargon term found in one message against previously reviewed messages. We included only four of the seven categories from Figure 1 in our analysis—abbreviations, technical terms, medicalized English, and medical vernacular. We did not include unnecessary synonyms, euphemisms, or judgmental jargon because we felt these were more subjective, culturally-influenced, difficult to operationalize, and more vulnerable to bias and interrater variability. Table 2 includes additional details about the four categories included in the analysis, including examples drawn from our data, and any specific exceptions to the decision rule.

### 2.5. Undefined Jargon

We determined whether an attempt was made to define, describe, explain or contextualize jargon terms in order to make them more understandable. Subjectively, we felt that many of these attempts were not likely helpful to the patient, but lacking a means for systematically judging the quality of such attempts, we coded any attempt as “defined.”

### 2.6. Jargon Counting

We counted a given undefined jargon term only once per message, because words are understood within the context in which they appear, and it is not known whether repeated use of an unfamiliar word within the context of a given written message increases the likelihood that the reader will accurately deduce its intended meaning. If a term was initially undefined but was later defined in the same message, we counted it as undefined. Like Miller and colleagues [11], we treated phrases with two or more jargon words that represented a unique entity (e.g., “glenohumeral joint” or “corticosteroid injection”) as a single jargon item. While none of the individual words in “out of pocket” are jargon, the phrase itself was counted as jargon because it represents a unique health-care-related entity. Unlike Miller and colleagues [11], we coded drug names (brand and generic) as jargon, based on the fact that drug names are clearly technical, are often not known by patients [36], and can be made more understandable with explanations and contextualization (e.g., the phrase, “Your amoxicillin should be ready to pick up” can be made less ambiguous by saying, “Your amoxicillin antibiotic pills for your sinus infection should be ready to pick up”).

### 2.7. Grade-Level Readability

We used the Flesch-Kincaid Grade Level calculator built into Microsoft Word version 2512 (Microsoft Corporation, Redmond, Washington, USA) to estimate the average readability of messages. Flesch-Kincaid is the most commonly used validated readability formula in health communication research [28]. It uses sentence length and number of syllables to estimate the educational grade level at which most readers would be expected to be able to read and understand a text. It does not account for jargon, and low-grade-level readability does not ensure understandability. Although data are limited, it has been suggested that the Flesch-Kincaid Grade Level readability formula is more stable and reliable for passages of 100 words or more because this reduces the likelihood that an outlier sentence will skew the results. We limited the readability analysis to messages of 100 words or longer only, in keeping with the approach of others [28].

### 2.8. Statistical Analysis

We compared pre- and post-training group means, percentages, and rates using two-tailed *t*-tests with a significance level of ≤0.05. For the jargon comparison, we tabulated raw numbers of jargon terms, counting each unique jargon term only once per message to derive the number of unique jargon terms. We then removed any terms that had been defined, to derive a unique undefined jargon list. We then divided this number by the total word count to arrive at a “unique undefined jargon rate” per 100 words of text. For secondary analyses, we calculated Benjamini–Hochberg adjusted *p*-values for a significance level q ≤ 0.05 to reduce the risk of Type I error when making multiple comparisons.

## 3. Results

All nine first-year residents contributed to electronic written messages sent in the pre- and post-training period. We did not collect demographic information about resident participants or patients receiving the messages. Comparing written messages sent in the 3 months prior to the training to those sent in the 3 months following the training, there were no statistically significant differences in the number of messages sent (210 before and 211 after training), average number of messages per resident, total word count, or average words per message. A total of 51 pre-training messages and 57 post-training messages contained 100 or more words and were included in the readability analysis, with mean grade-level readability estimates of 9.1 for both pre- and post-training messages (Table 3).

At baseline, residents used a large amount of unexplained jargon—7.2 terms per 100 words (average of 6.3 terms per message). For all subtypes of jargon combined, mean rates for unique undefined jargon decreased from 7.2 per 100 words before training to 6.3 per 100 words after training (*p* = 0.05) (Table 4), or from 6.3 to 5.5 jargon terms per message. Jargon rates declined for each of the jargon subtypes as well, though none were statistically significant. For all jargon subtypes, the percentage of undefined terms decreased after training, and the decreases for technical jargon and total jargon were statistically significant in unadjusted analyses. However, after adjusting for multiple comparisons, these decreases approached but did not achieve statistical significance (Table 4).

## 4. Discussion

To our knowledge, this is the first study to quantify physicians’ use of jargon in personalized written communication with patients and the first to report on the impact of a curriculum designed to reduce such use. Amongst a cohort of first-year family medicine residents, participation in a mandatory 80 min training on clear written communication was associated with a borderline significant (*p* = 0.05) reduction in the use of undefined jargon—from 7.2 to 6.3 jargon terms per 100 words—in secure electronic written communications with patients over 3 months of follow-up (Table 4). This 12.5% relative reduction from 6.3 to 5.5 undefined jargon terms per message is equivalent to nearly one fewer undefined jargon term per message on average. We believe the observed reduction in jargon use may be clinically significant in terms of patient satisfaction, as the use of just two undefined jargon terms by medical trainees was associated with lower ratings of professionalism by observers in a previous study [37]. While jargon is often poorly understood by patients [15,16,17,18,19,20,21,22], and this is believed to impede the understanding of health information [25], the clinical impact of unexplained jargon on patients’ ability to understand and act on health information (e.g., make appropriate health-related decisions) is not known, and additional outcome studies are needed in this area.

A particular strength of this study is that data were collected retrospectively. Participants were not aware at the time of writing that their messages would later be analyzed, thus eliminating the risk of performance bias, in which trainees change their behavior when aware they are being observed. As such, we believe that the observed behavior change (decreased use of unexplained jargon) may represent the development of a plain language “habit,” which, to our knowledge, has not been demonstrated previously in studies of either spoken or written communication. The 3-month post-training follow-up period is longer than in many educational outcome studies, adding strength to the interpretation that the observed communication may represent unprompted habits.

While the written communication workshop was Part 4 of a four-part longitudinal training (Table 1), we believe the change in jargon observed was primarily due to the written communication workshop itself. Part 1 (Intro to Health Literacy and Clear Communication) and Part 2 (Spoken Communication Best Practices Workshop) occurred approximately 6 months prior to the written communication workshop and 3 months before our baseline jargon assessment period. While Part 1 and/or Part 2 may have impacted residents’ use of jargon in the baseline assessment, it is less likely that they account for the reduction in jargon rates observed in the follow-up assessment. Part 3 (Numeracy), which occurred on the same day as the written communication workshop, also did not likely account for the reduction in jargon use, as it focused on expressing numerical information more clearly, rather than jargon or readability. Nonetheless, it is possible that one of these other parts of the curriculum could have served as a mediator or modifier, affecting the impact of the written communication workshop. Because a longitudinal curriculum is more challenging to deliver, future studies should determine whether a one-time written communication workshop can effectively reduce clinicians’ use of unexplained jargon.

We observed no change in the grade-level readability of written messages, which remained at the 9.1 grade level following the training. Experts recommend that patient information be written at the 5th–6th-grade level to improve accessibility [1,2]. In the only other study we are aware of that examined this issue, a curriculum for internal medicine residents and faculty improved the percentage of physicians whose personalized portions of After Visit Summaries were written at a 6th-grade level or lower from 30% before training to 68% after [27]. However, that study used ongoing feedback regarding the readability of personalized writing over a 6-month period, and the authors did not report whether their findings were statistically significant. During our training intervention, residents worked in teams with an explicit goal of reducing the grade-level readability of a rewritten message to a patient primarily by using one- or two-syllable words when possible and keeping sentences 15 words or shorter [38]. While readability levels were not recorded at that time, anecdotally, the teams achieved readability at or below the recommended 5th–6th-grade level. It is possible that our team-based training activity did not help the average resident develop lower-readability writing skills. Or, perhaps periodic behavioral cues or feedback are needed for developing lower-readability practices that persist up to 3 months following training [27].

A strength of our study is that we used a more inclusive definition of jargon than most others have, helping avoid information bias that might arise from non-empiric assumptions about which terms are more or less familiar or understandable to patients. This approach may, however, produce higher jargon rates than other approaches that attempt to distinguish between higher-level and lower-level jargon. Future studies should validate objective methods, such as those described by Wood and Gupta [13], for identifying “lower-level” jargon that can be confidently assumed to represent more desirable “plain language.”

This study has a number of other limitations: (1) We analyzed jargon at the message level, rather than the resident physician level, in this small cohort of nine residents. If one or more residents were significant outliers in their use of unexplained jargon, this could affect the generalizability of mean jargon rates, though it should not affect the validity of the observed change in mean rates. (2) Our single-cohort design did not include an unexposed control group. It is possible that the observed decrease in jargon was due to something other than the written communication workshop. For example, it is not known how residents’ clear communication skills may evolve over time. While we believe that the training caused the decrease in jargon use, we are careful to note that our results demonstrate an association and cannot determine causation. A randomized controlled or cross-over design study could provide more definitive evidence. (3) While we demonstrated reduced use of jargon associated with a written communication workshop, we were not able to determine whether other parts of the curriculum might be mediators or modifiers of this finding. (4) We did not analyze messages sent by patients, so we do not know which jargon terms may have been introduced by patients themselves. Patients are known to introduce jargon in written messages sent to clinicians through secure messaging platforms (e.g., “oxycodone HCL 5 mg tab,” “Parkinson’s,” “CT scan,” “bloodwork,” “EKG,” “referred [sent to]”) [39], and some authors have chosen not to count patient-initiated jargon [11]. If some of the jargon we documented had been previously introduced by the patient, our data may overestimate residents’ use of unexplained jargon. (5) We accepted any attempt to explain, define, or contextualize jargon terms as an explanation, without determining the effectiveness of such explanations. As such, our data may overestimate the amount of jargon that was adequately explained and the clinical significance of our results. (6) We do not know how well any of the written communication was understood by patients, or other patient-centered outcomes such as satisfaction, further limiting our ability to determine the clinical significance of reducing residents’ use of unexplained jargon. (7) We used a published taxonomy to categorize jargon types [34]. While this taxonomy has face validity, others have been proposed [40], and yet none have been validated. Jargon determination and classification thus remain subjective. Our use of a formal decision rule (Figure 1), however, helped minimize coding errors between categories. (8) We used an inclusive definition of jargon to help mitigate the subjective nature of jargon assessment. This captured both so-called higher- and lower-level jargon. While lower-level jargon may, in theory, be less problematic for patient understanding due to greater familiarity, we lacked a validated method for distinguishing between higher- and lower-level jargon. As such, the pre- and post-intervention jargon rates we observed may overestimate the amount of problematic jargon use. It is also possible that following the training, residents may have shifted from higher- to lower-level jargon, as the latter would align with greater use of plain language alternatives. If, for example, higher-level terms such as “hypertension” were replaced with lower-level terms like high “blood pressure,” our data may underestimate a shift in word choice toward plainer language. While Wood and Gupta proposed a method for delineating between higher- and lower-level jargon [13], future studies should attempt to validate such a method.

## 5. Conclusions

Physicians’ use of unnecessary and undefined jargon with patients has important implications for quality, safety, efficiency, patient-centeredness, satisfaction, and equity. Personalized written communication with patients, including electronic messaging through patient portals, as well as the provider-entered portions of care summaries, is a relatively underexplored area of provider–patient communication. Our study showed that resident physicians use a significant amount of unexplained jargon in written messages to patients, and that a brief educational intervention can reduce this use by fostering what we believe may be clear communication “habits,” something that few other studies have done [41]. More research is needed, however, to determine causality and whether there is a dose effect to the impact of plain language trainings such as ours, or whether booster trainings are needed to maintain plain language improvements over time. Our curriculum did not impact the grade-level readability of messages, and this remains an area worthy of additional study. Assessing jargon content in written messages is labor-intensive, limiting its utility as a tool for awareness-raising, skill and competency tracking, or curriculum evaluation. Advances in artificial intelligence, however, offer promising opportunities to improve our capacity to evaluate trainees’ and practicing clinicians’ written communication with patients. Assessing residents’ written communication periodically over the course of the training program and using such assessments as part of a longitudinal clear communication curriculum, for example, may help ensure that graduates develop more effective communication skills. Ultimately, however, researchers need to determine whether reducing clinicians’ use of unexplained jargon in personalized written communication improves patients’ experiences in terms of understanding and ability to act on health-related information, as well as satisfaction with care.

## Figures and Tables

**Table 1:** Required health literacy and clear communication longitudinal curriculum for first-year Family Medicine residents at Oregon Health & Science University, 2023–2024.

	Session Title	Learning Objectives	Description
**Part 1** (June 2023)	Intro to Health Literacy and Clear Communication	Describe the importance of literacy to health communication for all patients Define the term “health literacy” Discuss the prevalence of low health literacy and how all patients are at risk for communication errors Describe reasons why clinicians cannot reliably tell who has low health literacy in any given medical encounter Describe reasons why a “universal precautions” approach to health communication is needed when working with all patients List at least five concrete steps you can take to promote a “universal precautions” approach	2 h interactive didactic
**Part 2** (July 2023)	Spoken Communication Best Practices Workshop	Demonstrate the ability to use plain language and avoid unnecessary or undefined jargon in spoken patient communication Demonstrate the ability to avoid information overload in spoken patient communication Demonstrate the ability to use teach-back to confirm that clear communication has occurred	2 h skill-building workshop includes didactics, plus role-play practice discussing test results using plain language, and using teach-back
**Part 3** (Feb 2024)	Numeracy: Writing Easy-To-Adhere-To Prescriptions *	Recognize the prevalence of low numeracy among patient populations Create easy-to-adhere-to prescription instructions	40 min interactive, didactic, and small team prescription-writing activity *
**Part 4** (Feb 2024)	Written Communication Best Practices Workshop	Describe best practices for creating personalized written health messages for patients and caregivers (includes avoiding jargon, writing at a 5th–6th-grade readability level, identifying the purpose of the writing, putting key information at the beginning, and avoiding information overload) Create personalized written health messages that improve understandability and actionability	80 min skill-building workshop includes didactic review of best practices, plus rewriting test result notes for improved readability, understandability, and actionability *

* In 2023–2024, the usual 2 h Parts 3 and 4 were collapsed into a single 2 h session (40 min on numeracy specific to prescription-writing, and 80 min on written communication) due to a weather event.

**Table 2:** Modified * seven-category jargon taxonomy [34] with examples from residents’ written messages to patients.

Jargon Category	Description	Examples ^†^	Notes and Exceptions
Technical terms (classic jargon)	Anatomy, disease names, *drug names*, job titles, microbes, symptom names, tests and procedures, therapies	Bilirubin Colonoscopy Creatinine Gastroenterology Levothyroxine Lung Palpitations Plantar fascia Phlebotomy (lab) Triglycerides	Easily recognizable external anatomy (e.g., eyes, head, hips) was not coded as jargon; internal anatomy (e.g., heart, liver, lungs) was coded as jargon because internal anatomy is poorly understood (Taylor et al., 2018) [35]
Abbreviations, acronyms, truncations, or *shorthand* *symbols*	Acronyms for technical *and non-technical* terms, abbreviations and shorthand, symbols	< CBC COPD CT scan GI Lab mg Ortho Script (prescription) Snafu	Time (i.e., AM/PM), states (e.g., OR [Oregon]), directions (e.g., SW), and weblinks were not coded as jargon; “lab” and “exam” were coded as truncations but could also be medical vernacular
Medical vernacular	*Categories of technical terms*, descriptions of symptoms, syndromes, health-related phenomena, and *neologisms*	Antibiotics Blood pressure Chronic Diagnostics eConsult Electrolytes Follow-up [noun] Imaging Mental health Over-the-counters Procedure Provider Referral Side effects	“Schedule,” “appointment,” and “visit” were not coded as jargon due to widespread use in other contexts; “blood pressure” and “pill” were coded as jargon despite being preferred plain language alternatives to “hypertension” and “tablet,” for example
Medicalized English	Words with different meanings inside and outside of health-related contexts	Covered Drawn Indicated Negative Positive Stable Stool Study	
Unnecessary synonyms	Less common terms used when a more familiar one would suffice	Cumbersome Dissipate Expediency	Not included in analysis
Euphemisms *and idioms*	Vague phrasing or colloquialisms that attempt to soften language but may reduce clarity	Not a good candidate for surgery Slipping through the cracks	Not included in analysis
Judgmental jargon	Phrases that appear derogatory or suggest bias	[None observed]	Not included in analysis

* Portions in italics represent significant change from Pitt and Hendrickson [34]. ^†^ Examples taken from electronic messages sent to patients in the present study.

**Table 3:** Residents’ written messages before and after communication training.

	Pre-Training	Post-Training	Total	*p*
Number of resident authors	9	9	9	-
Total number of messages (all residents)	210	211	421	-
Mean (SD) number of messages per resident	23.3 (18.4)	23.4 (15.6)	23.4 (16.6)	0.989
Total word count, all messages	18,192	18,562	36,754	-
Mean (SD) words per message	86.6 (81.3)	88.0 (75.5)	87.3 (78.4)	0.860
Number of messages with ≥100 words	51	57	108	-
Mean (SD) grade-level readability of messages with ≥100 words *	9.1 (2.9) [95% CI 8.7–9.5]	9.1 (2.6) [95% CI 8.7–9.5]	9.1 (2.7) [95% CI 8.8–9.4]	0.896

* Readability estimated using the Flesch-Kincaid Grade Level readability tool in Microsoft Word version 2512.

**Table 4:** Jargon in residents’ written messages before and after communication training by jargon subtype.

Subtype	Item	Pre-Training (n = 210)	Post-Training (n = 211)	*p*	Adjusted *p* ^†^
Technical	Number of technical terms (all residents)	481	483	-	-
	Number of unique * technical terms (all residents)	414	418	-	-
	Number (%) of unique * undefined technical terms	399/414 (96.4%)	386/418 (92.3%)	0.01	0.07
	Mean (SD) number of unique * undefined technical terms per message	399/210 = 1.9 (2.4)	386/211 = 1.8 (2.1)	0.75	0.81
	Mean (SD) unique * undefined technical jargon rate per 100 words	2.2 (2.0) [95% CI 1.9–2.5]	2.1 (1.8) [95% CI 1.9–2.3]	0.29	0.51
Acronyms/abbreviations	Number of abbreviated terms (all residents)	324	256	-	-
	Number of unique * abbreviated terms (all residents)	270	220	-	-
	Number (%) of unique * undefined abbreviated terms	260/270 (96.3%)	211/220 (95.9%)	0.82	0.82
	Mean (SD) number of unique * undefined abbreviation terms per message	260/210 = 1.2 (1.7)	211/211 = 1.0 (1.7)	0.15	0.35
	Mean (SD) unique * undefined abbreviated jargon rate per 100 words	1.4 (1.8) [95% CI 1.2–1.6]	1.1 (1.8) [95% CI 0.9–1.3]	0.07	0.33
Medical vernacular	Number of vernacular terms (all residents)	579	541	-	-
	Number of unique * vernacular terms (all residents)	515	469	-	-
	Number (%) of unique * undefined vernacular terms	512/515 (99.4%)	465/469 (99.2%)	0.61	0.71
	Mean (SD) number of undefined unique* vernacular terms per message	512/210 = 2.4 (2.4)	465/211 = 2.2 (1.9)	0.27	0.51
	Mean (SD) unique * undefined vernacular jargon rate per 100 words	2.8 (2.2) [95% CI 2.5–3.1]	2.5 (2.2) [95% CI 2.2–2.8]	0.36	0.56
Medicalized English	Number of medicalized English terms (all residents)	151	131	-	-
	Number of unique * medicalized English terms (all residents)	131	118	-	-
	Number (%) of unique * undefined medicalized English terms	131/131 (100.0%)	116/118 (98.3%)	0.14	0.35
	Mean (SD) number of unique * undefined medicalized English terms per message	131/210 = 0.6 (1.2)	116/211 = 0.5 (0.9)	0.46	0.64
	Mean (SD) unique * undefined medicalized English rate per 100 words	0.7 (1.3) [95% CI 0.5–0.9]	0.6 (1.1) [95% CI 0.4–0.8]	0.60	0.71
All forms of jargon	Number of jargon terms—all types (all residents)	1535	1411	-	-
	Number of unique * jargon terms—all types (all residents)	1330	1225	-	-
	Number (%) of unique * undefined jargon terms—all types	1302/1330 (97.9%)	1178/1225 (96.2%)	0.01	0.07
	Mean (SD) number of unique * undefined jargon terms per message— all types	1302/210 = 6.2 (2.12)	1178/211 = 5.6 (1.80)	0.12	0.35
	Mean (SD) unique * undefined jargon rate per 100 words—all jargon types	7.2 (2.0) [95% CI 6.9–7.5]	6.3 (2.0) [95% CI 6.0–6.6]	0.05	n/a ^‡^

* Unique terms: jargon terms appearing more than once in the same message are counted only once. ^†^ Benjamini–Hochberg adjusted *p*-values for multiple comparisons at a False Discovery Rate of q < 0.05 for all secondary outcomes. ^‡^ *p*-value not adjusted for primary outcome measure.

## Data Availability

The data presented in this study are available on request from the corresponding author to help protect the privacy of residents and patients whose communications were used.
